# Supplementary material for: Dietary Supplement Intake and Fecundability in a Singapore Preconception Cohort Study
Source: Nutrients. 2022 Dec 1;14(23):5110. doi: 10.3390/nu14235110 (PMC9739604; doi:10.3390/nu14235110)
Supplement: Supplementary file 1 [file nutrients-14-05110-s001.zip › nutrients-2035961-supplementary.pdf]

Table S1. Characteristics of women according to inclusion status from the S-PRESTO study, 2015-2018 (n=1032).

**Characteristics**	**Excluded (n=124)**	**Included (n=908)**	**p[a]**
Age			0.583
<35 years	108 (87.1)	774 (85.2)	
≥35 years	16 (12.9)	134 (14.8)	
Ethnicity			0.638
Chinese	89 (71.8)	654 (72.0)	
Malay	16 (12.9)	143 (15.7)	
Indian	13 (10.5)	82 (9.0)	
Mix	6 (4.8)	29 (3.2)	
Parity			0.171
0	60 (58.8)	596 (65.6)	
≥1	42 (41.2)	312 (34.4)	
Highest education			0.283
Below tertiary	47 (42.3)	337 (37.1)	
Tertiary and above	64 (57.7)	571 (62.9)	
Body mass index[b]			0.865
Underweight <18.5 kg/m^2	8 (8.3)	76 (8.4)	
Normal 18.5-22.9 kg/m^2	42 (43.8)	420 (46.3)	
Overweight 23-27.4 kg/m^2	29 (30.2)	238 (26.2)	
Obese ≥27.5 kg/m^2	17 (17.7)	174 (19.2)	
Cycle regularity			0.005
Regular	58 (52.3)	597 (65.7)	
Irregular	53 (47.7)	311 (34.3)	
Cycle length, days	30.0 (28.5-33.5)	29.5 (29.0-32.5)	0.848
Smoking exposure			0.358
No	75 (72.8)	698 (76.9)	
Yes	28 (27.2)	210 (23.1)	
Alcohol intake			0.663
No	68 (54.8)	479 (52.8)	
Yes	56 (45.2)	429 (47.2)	
Unhealthful plant-based diet index			0.940
Tertile 1 ≤41	36 (35.6)	331 (36.5)	
Tertile 2 >41 to ≤47	34 (33.7)	290 (31.9)	
Tertile 3 >47	31 (30.7)	287 (31.6)	
Total daily energy intake, kcal/d	2047 (1651-2562)	1945 (1567-2385)	0.116

Data are presented in number (percentage) for categorical variables and median (25th – 75th percentile) for continuous variables. Total sample size of excluded women does not always equal to 124 due to missing data. S-PRESTO, Singapore PREconception Study of long-Term maternal and child Outcomes.
[a]Based on Pearson's chi-squared test for categorical variables and Mann-Whitney test for continuous variables.
[b]Classified based on cut-offs for Asian populations [42].

Table S2. Associations between supplement intake status and fecundability in women with pregnancy attempts of ≤3, ≤6 and ≤12 months at study entry from the S-PRESTO study, 2015-2018.

	**Attempted time to conceive at study entry**					
	**≤3 months (n=527)**		**≤6 months (n=653)**		**≤12 months (n=774)**	
**Type of supplements**	**FR**	**95% CI**	**FR**	**95% CI**	**FR**	**95% CI**
Supplement intake status						
No supplements	1.00	(Ref.)	1.00	(Ref.)	1.00	(Ref.)
Any supplement	1.29	0.98, 1.68	1.25	0.98, 1.60	1.29	1.02, 1.64
Folic acid						
Non-user	1.00	(Ref.)	1.00	(Ref.)	1.00	(Ref.)
User	1.32	1.03, 1.68	1.26	1.01, 1.58	1.31	1.05, 1.62
Folic acid type						
Non-user	1.00	(Ref.)	1.00	(Ref.)	1.00	(Ref.)
Single vitamin	1.41	1.02, 1.95	1.29	0.96, 1.72	1.32	1.01, 1.73
Multivitamin	1.18	0.86, 1.62	1.20	0.90, 1.61	1.22	0.92, 1.62
Single vitamin and multivitamin	1.57	0.97, 2.55	1.38	0.90, 2.12	1.53	1.03, 2.28
Fish oil						
Non-user	1.00	(Ref.)	1.00	(Ref.)	1.00	(Ref.)
User	1.01	0.75, 1.37	1.00	0.76, 1.31	1.03	0.80, 1.33
Evening primrose oil						
Non-user	1.00	(Ref.)	1.00	(Ref.)	1.00	(Ref.)
User	0.41	0.18, 0.92	0.54	0.29, 1.01	0.54	0.30, 0.99
Iron						
Non-user	1.00	(Ref.)	1.00	(Ref.)	1.00	(Ref.)
User	1.16	0.88, 1.53	1.19	0.93, 1.52	1.21	0.95, 1.53
Zinc						
Non-user	1.00	(Ref.)	1.00	(Ref.)	1.00	(Ref.)
User	1.11	0.83, 1.47	1.14	0.88, 1.47	1.16	0.91, 1.49
Selenium						
Non-user	1.00	(Ref.)	1.00	(Ref.)	1.00	(Ref.)
User	1.14	0.84, 1.54	1.16	0.88, 1.53	1.22	0.94, 1.58
Iodine						
Non-user	1.00	(Ref.)	1.00	(Ref.)	1.00	(Ref.)
User	1.23	0.91, 1.66	1.26	0.96, 1.65	1.29	1.00, 1.67
Vitamin B6						
Non-user	1.00	(Ref.)	1.00	(Ref.)	1.00	(Ref.)
User	1.13	0.87, 1.48	1.15	0.90, 1.47	1.17	0.93, 1.48
Vitamin B12						
Non-user	1.00	(Ref.)	1.00	(Ref.)	1.00	(Ref.)
User	1.19	0.91, 1.55	1.18	0.92, 1.50	1.19	0.94, 1.50
Vitamin C						
Non-user	1.00	(Ref.)	1.00	(Ref.)	1.00	(Ref.)
User	1.21	0.94, 1.56	1.17	0.93, 1.48	1.18	0.95, 1.48
Vitamin D						
Non-user	1.00	(Ref.)	1.00	(Ref.)	1.00	(Ref.)
User	1.12	0.85, 1.47	1.14	0.89, 1.47	1.17	0.93, 1.49
Vitamin E						
Non-user	1.00	(Ref.)	1.00	(Ref.)	1.00	(Ref.)
User	1.05	0.79, 1.40	1.10	0.85, 1.42	1.14	0.89, 1.46

Data were analysed using the discrete-time proportional hazards model, adjusting for age, ethnicity, education, parity, body mass index, cycle regularity, smoking exposure, alcohol intake, unhealthful plant-based diet index and total daily energy intake. S-PRESTO, Singapore PREconception Study of long-Term maternal and child Outcomes; FR, fecundability ratio; CI, confidence interval; Ref., reference.

Table S3. Associations between supplement intake status and fecundability in women without polycystic ovarian syndrome (PCOS) or using singleton live birth as the outcome measure from the S-PRESTO study, 2015-2018.

	**Without PCOS (n=898)**		**Singleton live birth as outcome (n=908)**	
**Type of supplements**	**FR**	**95% CI**	**FR**	**95% CI**
Supplement intake status				
No supplements	1.00	(Ref.)	1.00	(Ref.)
Any supplement	1.32	1.05, 1.66	1.37	1.08, 1.75
Folic acid				
Non-user	1.00	(Ref.)	1.00	(Ref.)
User	1.29	1.05, 1.60	1.34	1.08, 1.67
Folic acid type				
Non-user	1.00	(Ref.)	1.00	(Ref.)
Single vitamin	1.30	1.00, 1.68	1.34	1.03, 1.76
Multivitamin	1.25	0.95, 1.65	1.23	0.92, 1.65
Single vitamin and multivitamin	1.41	0.95, 2.10	1.67	1.12, 2.47
Fish oil				
Non-user	1.00	(Ref.)	1.00	(Ref.)
User	1.00	0.78, 1.28	0.98	0.76, 1.28
Evening primrose oil				
Non-user	1.00	(Ref.)	1.00	(Ref.)
User	0.58	0.32, 1.03	0.66	0.38, 1.15
Iron				
Non-user	1.00	(Ref.)	1.00	(Ref.)
User	1.19	0.95, 1.50	1.23	0.97, 1.56
Zinc				
Non-user	1.00	(Ref.)	1.00	(Ref.)
User	1.14	0.89, 1.44	1.21	0.94, 1.55
Selenium				
Non-user	1.00	(Ref.)	1.00	(Ref.)
User	1.18	0.92, 1.53	1.21	0.93, 1.58
Iodine				
Non-user	1.00	(Ref.)	1.00	(Ref.)
User	1.29	1.00, 1.66	1.29	0.99, 1.68
Vitamin B6				
Non-user	1.00	(Ref.)	1.00	(Ref.)
User	1.18	0.94, 1.48	1.20	0.95, 1.53
Vitamin B12				
Non-user	1.00	(Ref.)	1.00	(Ref.)
User	1.20	0.95, 1.50	1.22	0.97, 1.55
Vitamin C				
Non-user	1.00	(Ref.)	1.00	(Ref.)
User	1.18	0.95, 1.46	1.24	0.99, 1.55
Vitamin D				
Non-user	1.00	(Ref.)	1.00	(Ref.)
User	1.14	0.90, 1.44	1.17	0.91, 1.49
Vitamin E				
Non-user	1.00	(Ref.)	1.00	(Ref.)
User	1.10	0.86, 1.40	1.17	0.91, 1.50

Data were analysed using the discrete-time proportional hazards model, adjusting for age, ethnicity, education, parity, body mass index, cycle regularity, smoking exposure, alcohol intake, unhealthful plant-based diet index and total daily energy intake. S-PRESTO, Singapore PREconception Study of long-Term maternal and child Outcomes; PCOS, polycystic ovarian syndrome; FR, fecundability ratio; CI, confidence interval; Ref., reference.

Table S4. Serum/plasma micronutrient levels of women based on their respective supplement intake status for folic acid, vitamins B6, B12, D, and E from the S-PRESTO study, 2015-2018.

Serum/plasma micronutrients	Total	Non-user	User	p[a]
Folic acid, nmol/L	28.7 (16.9-54.4)	18.1 (12.8-25.2)	51.0 (32.5-79.0)	<0.001
Vitamin B6 (pyridoxal phosphate), nmol/L	54.4 (39.9-85.3)	48.6 (37.0-68.9)	109.5 (59.4-223.0)	<0.001
Vitamin B12, pmol/L	245.0 (190.0-313.0)	230.5 (180.0-287.0)	302.0 (230.5-385.0)	<0.001
Vitamin D3, nmol/L	52.2 (38.3-62.9)	50.1 (36.7-60.1)	58.1 (49.7-68.2)	<0.001
Vitamin E (alpha-tocopherol), µmol/L	32.5 (28.7-37.6)	31.8 (28.4-36.4)	35.2 (30.3-40.3)	<0.001

Data are presented in median (25th – 75th percentile). S-PRESTO, Singapore PREconception Study of long-Term maternal and child Outcomes.

[a]Based on Mann-Whitney test.

Table S5. Associations between serum/plasma micronutrient levels and fecundability in women from the S-PRESTO study, 2015-2018.

Serum/plasma micronutrients	n (%)	FR	95% CI
Folic acid, nmol/L			
Tertile 1 ≤20.0	294 (33.4)	1.00	(Ref.)
Tertile 2 >20.0 to ≤44.6	294 (33.4)	1.25	0.96, 1.62
Tertile 3 >44.6	292 (33.2)	1.47	1.12, 1.93
Vitamin B6 (pyridoxal phosphate), nmol/L			
Tertile 1 ≤43.7	292 (33.3)	1.00	(Ref.)
Tertile 2 >43.7 to ≤71.7	293 (33.4)	0.99	0.76, 1.28
Tertile 3 >71.7	291 (33.2)	1.04	0.80, 1.35
Vitamin B12, pmol/L			
Tertile 1 ≤208.0	303 (34.4)	1.00	(Ref.)
Tertile 2 >208.0 to ≤284.0	284 (32.3)	1.23	0.95, 1.59
Tertile 3 >284.0	293 (33.3)	1.26	0.97, 1.63
Vitamin D3, nmol/L			
Tertile 1 ≤44.0	293 (33.4)	1.00	(Ref.)
Tertile 2 >44.0 to ≤58.4	292 (33.3)	0.96	0.72, 1.28
Tertile 3 >58.4	292 (33.3)	1.09	0.82, 1.46
Vitamin E (alpha-tocopherol), µmol/L			
Tertile 1 ≤29.7	294 (33.5)	1.00	(Ref.)
Tertile 2 >29.7 to ≤35.7	291 (33.2)	0.87	0.68, 1.11
Tertile 3 >35.7	292 (33.3)	0.87	0.67, 1.11

Data were analysed using the discrete-time proportional hazards model, adjusting for age, ethnicity, education, parity, body mass index, cycle regularity, smoking exposure, alcohol intake, unhealthful plant-based diet index and total daily energy intake. S-PRESTO, Singapore PREconception Study of long-Term maternal and child Outcomes; FR, fecundability ratio; CI, confidence interval; Ref., reference.

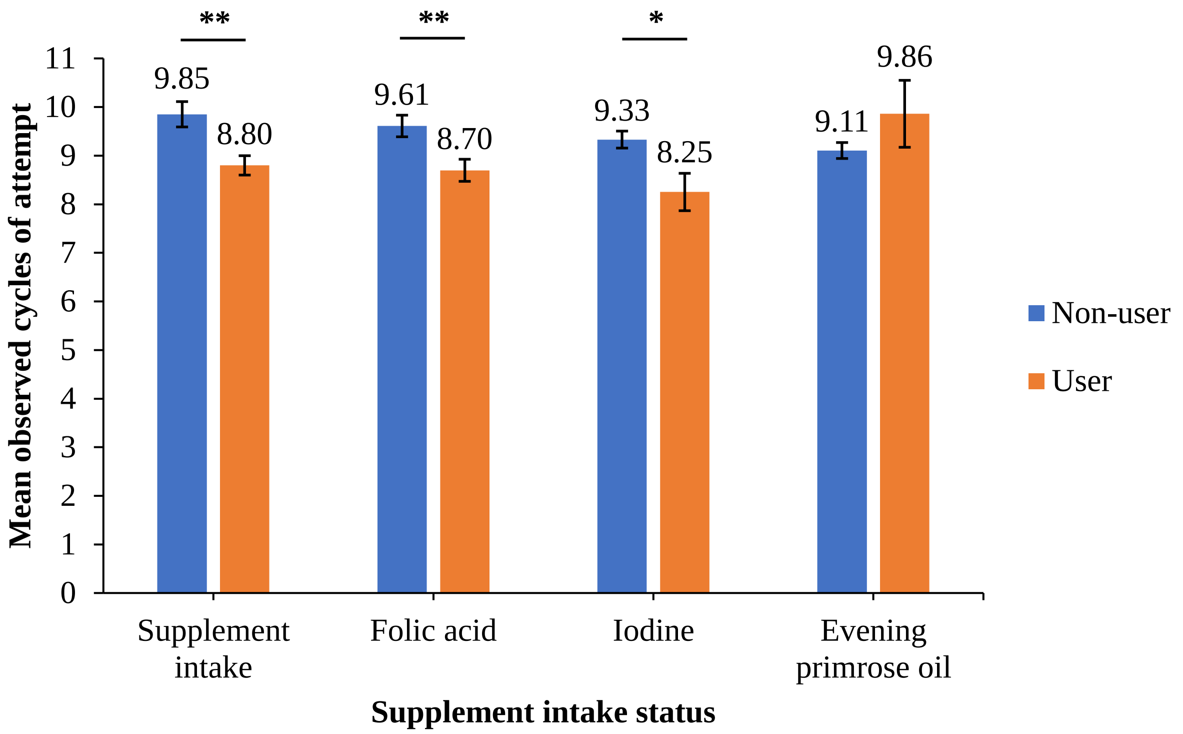

Figure S1. Bar chart showing the mean observed cycles of attempt by supplement intake status from the S-PRESTO study, 2015-2018 (n=908). Error bars denote ± standard error of mean. Based on unpaired t-test, * denotes p <0.05 and ** denotes p < 0.01. S-PRESTO, Singapore PREconception Study of long-Term maternal and child Outcomes.
